# Whole-Plant Metabolic Allocation Under Water Stress

**DOI:** 10.3389/fpls.2018.00852

**Published:** 2018-06-25

**Authors:** Fabiane M. Mundim, Elizabeth G. Pringle

**Affiliations:** ^1^Department of Biology, University of Nevada, Reno, Reno, NV, United States; ^2^Program in Ecology, Evolution and Conservation Biology, University of Nevada, Reno, Reno, NV, United States

**Keywords:** abiotic stress, biotic stress, drought, growth-defense trade-off, herbivory, multiple stresses, resource allocation, roots

## Abstract

Trade-offs between plant growth and defense depend on environmental resource availability. Plants are predicted to prioritize growth when environmental resources are abundant and defense when environmental resources are scarce. Nevertheless, such predictions lack a whole-plant perspective—they do not account for potential differences in plant allocation above- and belowground. Such accounting is important because leaves and roots, though both critical to plant survival and fitness, differ in their resource-uptake roles and, often, in their vulnerability to herbivores. Here we aimed to determine how water availability affects plant allocation to multiple metabolic components of growth and defense in both leaves and roots. To do this, we conducted a meta-analysis of data from experimental studies in the literature. We assessed plant metabolic responses to experimentally reduced water availability, including changes in growth, nutrients, physical defenses, primary metabolites, hormones, and other secondary metabolites. Both above- and belowground, reduced water availability reduced plant biomass but increased the concentrations of primary metabolites and hormones. Importantly, however, reduced water had opposite effects in different organs on the concentrations of other secondary metabolites: reduced water increased carbon-based secondary metabolites in leaves but reduced them in roots. In addition, plants suffering from co-occurring drought and herbivory stresses exhibited dampened metabolic responses, suggesting a metabolic cost of multiple stresses. Our study highlights the needs for additional empirical studies of whole-plant metabolic responses under multiple stresses and for refinement of existing plant growth-defense theory in the context of whole plants.

## Introduction

Plants experience many forms of stress, from both the abiotic and the biotic environment. As sessile organisms, plants have evolved various physiologic and metabolic responses to individual stresses, but the nature of such responses strongly depends on whether and how stresses co-occur in the plant’s environment (Atkinson and Urwin, 2012; Suzuki et al., 2014; Nguyen et al., 2016). In particular, the co-occurrence of resource limitation and herbivory can steepen the trade-off between growth and defense by altering both the availability of chemical precursors and the strategic value of defense (Herms and Mattson, 1992; Mole, 1994; Donaldson et al., 2006). The strategic value of defense (*i.e.*, optimal defense) should depend on the cost of defense traits, the value of the tissue, and the risk of attack from herbivores (McKey, 1974; Rhoades, 1979). Although roots are the first responders to many kinds of stress (Brunner et al., 2015; Weemstra et al., 2016), work to date on growth-defense trade-offs and optimal defense has focused mostly aboveground, on shoots and leaves, rather than on the whole plant (van Dam, 2009). There are still few predictions about how simultaneous abiotic and biotic stresses should drive whole-plant allocation strategies.

Water availability is a central resource affecting plant fitness. Predicted increases in the frequency of extreme precipitation events under ongoing global climate change (Bates et al., 2008; Donat et al., 2016) threaten reliable sources of water for terrestrial ecosystems (Easterling et al., 2000; Weltzin et al., 2003). Plants experiencing drought or flooding can adjust their morphology to optimize water uptake by the roots while decreasing the rate of photosynthesis by the leaves, thereby changing the production of growth and defense metabolites (Koricheva et al., 1998; Grant et al., 2005; Nicotra et al., 2007; Kleine and Mueller, 2014). Changes in biochemistry under water stress can determine plant physiology and performance, including the fitness-defining production of flowers and seeds (Taiz and Zeiger, 1998). Despite the potentially vital role of water availability in driving trade-offs between growth and defense, few studies to date have evaluated whole-plant metabolic responses to water stress in combination with herbivory stress, either experimentally induced or inferred from the plant’s production of defensive secondary metabolites. Because plant chemistry links multi-species trophic interactions with biogeochemical cycles, determining how plant chemistry responds to changes in precipitation may be critical to determining the response of entire ecosystems (Hunter, 2016).

Plant responses to water stress can affect the concentration, composition, and distribution of both primary and secondary metabolites. Plant primary metabolites, such as amino acids, enzymes, and carbohydrates, maintain life processes and facilitate growth (Díaz et al., 2004). Secondary metabolites allow plants to adapt to their environments by defending them against abiotic stresses, pathogens, and herbivores (Agrawal, 2007; Ramakrishna and Ravishankar, 2011). A plant’s response to stress typically begins with an elaborate signaling network, with frequent crosstalk between primary and secondary metabolic pathways (Robert-Seilaniantz et al., 2011; Atkinson and Urwin, 2012; Bonaventure, 2014; Suzuki et al., 2014; Jacobo-Velázquez et al., 2015). Signaling pathways can also be shared between responses to different forms of stress, including between biotic and abiotic stresses (Santner and Estelle, 2009; Robert-Seilaniantz et al., 2011; Atkinson and Urwin, 2012; Denancé et al., 2013; Nguyen et al., 2016). Changes in the quantity and composition of signal molecules induced by simultaneous stresses may in fact allow plants to alter their physiologies and metabolic mechanisms to cope with multiple stresses at once (Krasensky and Jonak, 2012). For example, abscisic acid (ABA) and jasmonic acid (JA) hormone signaling regulate plant responses to both drought and foliar insect herbivores (Pieterse et al., 2012; Berens et al., 2017). The cost of plant responses to both stresses may be reduced by this overlap (Mittler, 2006; Nguyen et al., 2016). In other cases, different stresses elicit opposing reactions. For example, salicylic acid (SA) and ABA/JA signaling pathways are commonly antagonistic to one another (Pieterse et al., 2012; Berens et al., 2017). Elevated SA signaling in response to biotrophic pathogens is often correlated with reduced ABA/JA signaling and decreased resistance to drought and insect herbivores (Zarate et al., 2007; Suzuki et al., 2014).

Changes in plant chemistry in response to water stress, although measured less frequently in ecological studies than changes in biomass and reproduction (but see Koricheva et al., 1998), will strongly affect the surrounding ecological community via direct and indirect trophic interactions (Hunter, 2016). These interactions can then feed back to affect plant chemistry and nutrient cycles. Such complex interactions initiate not only in leaves, but also in roots. Plant metabolic responses to water stress are also likely to differ between leaves and roots (Parker et al., 2012). Because water is sensed by the roots, root metabolic allocation under water stress is probably critical to defining whole-plant responses (Wilkinson and Davies, 2010; Basu et al., 2016). Although water stress often appears correlated with differences in herbivore pressure aboveground (White, 1969; Mattson and Haack, 1987; Huberty and Denno, 2004), very little is known about how water stress affects the susceptibility of roots to attack.

In this study, we assemble a meta-analytic database to synthesize our knowledge so far of: (1) how whole plants respond metabolically to reduced water; and (2) if and how these responses differ when plants suffer from co-occurring herbivory stress. To obtain sufficient studies for our analysis, we examined any study that reduced water compared to controls (see Results for details). For convenience, we use “drought” interchangeably with reduced water, a convention that is consistent conceptually, although not in operational detail, with the formal hydrological definition of drought (Mishra and Singh, 2010). Specifically, we test the hypotheses that: (1) leaves and roots produce different metabolic responses under reduced water; (2) reduced water negatively affects nutrient concentrations in both leaves and roots (He and Dijkstra, 2014); (3) reduced water increases the concentration of primary metabolites in both leaves and roots (Chaves et al., 2003), but the effects on composition vary between above- and belowground organs; (4) changes in secondary metabolites under reduced water are related to distinct growth-defense trade-offs in roots and leaves because the higher relative growth of roots alone can mitigate the effects of drought; and (5) the cost of co-occurring drought and herbivory stresses is mitigated by the overlap in ABA/JA defense signaling pathways.

## Materials and Methods

### Study Search and Data Collection

We compiled the database by conducting a key-word search in the Web of Science (ISI) in September 2017. We considered all resulting peer-reviewed studies with no date restrictions from searches using the terms “secondary metabolites or compounds”, “chemical compounds or defens^∗^”, “plant or leav^∗^ or root^∗^” and “herbivor^∗^ or insect or parasitoid” as a topic in all possible factorial combinations, but always with “water or precipitation or drought” as the title category. This initial search resulted in 1,475 studies. Our analysis did not include book chapters, graduate theses, or unpublished data. We attempted to analyze the effects of increased water, or flooding, stress on plant metabolism as well, but found too few studies that had addressed this alternative water stress to be confident in our results (data not shown).

To be included in the analysis, each study had to meet three criteria. First, water or precipitation had to have been manipulated experimentally, *i.e.*, the study had to have both control and treatment levels of water. Observational studies comparing plants growing in variable natural conditions were thus excluded from the analysis. Second, at least one secondary chemical metabolite (including hormones) had to have been reported. Third, studies had to provide each of the following variables (directly or indirectly): means, measure of variance (SD or SE), and the sample sizes of the control and the treatments. When this statistical information was not reported in the text, we extracted these values from the data using “GetData Graph Digitizer” (v2.24; Fedorov, 2002). Unspecific error bars were assumed to show standard error.

We also followed two rules when collecting the data from each study: (1) When a single study presented results for several plant species and response variables, we included them all. (2) When plant species were subjected to a gradient of water treatments or several treatments in a factorial design, we chose the control, and the lowest value of water as “drought.” Although an imperfect proxy for real-world drought, this definition allows us to begin to address how plants may respond metabolically to changes in precipitation. Results from factorial designs in which water treatment was in combination with another treatment (*e.g.*, low nutrient, shade) were excluded.

We investigated plant metabolic responses to drought alone and to drought combined with herbivory. We grouped the response variables into six overarching groups: (1) growth, including root and/or shoot weight (dry or fresh); (2) nutrients, including nitrogen (N), phosphorus (P), and potassium (K) concentrations from whole plants, roots, and/or shoots; (3) physical traits, including lignin, specific leaf area, and root length; (4) primary metabolites, including leaf soluble sugars (mono-, di-, and trisaccharides), complex carbohydrates (starch and/or total non-structural carbohydrates), vitamins, amino acids, and enzymes; (5) hormones, including abscisic acid (ABA) and jasmonic acid (JA), which are important for plant drought and herbivory responses; and (6) other secondary metabolites, including both carbon- and nitrogen-based secondary metabolites (*i.e.*, flavonoids, phenolics, tannins, terpenoids, volatiles, alkaloids, stilbenes, and glucosinolates) from roots and shoots. Although the ‘volatiles’ mainly comprised monoterpenes and sesquiterpenes, we separated them from “terpenoids” due to the method of extraction: volatiles were extracted from head-space collections, whereas terpenoids were extracted from ground leaves. We also collected the following additional information from each study: plant species, study location (greenhouse, growth chamber, or field), type of reduced water treatment, herbivore species, plant part attacked by herbivores (leaf, roots, or both), herbivore feeding type (*i.e.*, chewing or sap-sucking), and the indirect effect on parasitoids.

### Statistical Analyses

For each study and response variable, we estimated the mean effect size of a plant subjected to water treatments using Hedges’ *d* statistic (Koricheva et al., 2013). We estimated the magnitude of the treatment effect (effect size, *d*) by calculating the difference between the treatment and control estimated means, adjusted by their sample sizes and standard deviation, and weighted by a correction term (Gurevitch et al., 2001; Koricheva et al., 2013). We use Hedges’ *d* because it is not affected by unequal sampling variances, and it includes a correction factor for small sample sizes (Gurevitch et al., 2001). We calculated the mean effect size and confidence interval (CI) for each class of response variable. The water treatment was considered to have a statistically significant effect when the 95% CI of the variable did not overlap zero. A positive effect indicates that the drought treatment increased the amount of a given plant trait, whereas a negative effect indicates that the water treatment decreased the amount of a given plant trait.

To test if there was variation among studies beyond that due to sampling error, we used the model heterogeneity statistic (Q*_M_*, also known as heterogeneity between groups Q*_B_*). Q*_M_* describes the amount of heterogeneity that can be explained by the model (Gurevitch et al., 2001; Koricheva et al., 2013). Here this means that if Q*_M_* for a given plant trait is significant (*P* < 0.05), some of the variance can be explained by the water treatment. We calculated Q*_M_* using the Q statistic and then compared against the Q*_E_* (unexplained heterogeneity, also known as Q*_Error_*) using a chi-squared distribution.

We conducted all analyses using the R statistical programming language (v3.2.4; R Core Team, 2016) with the package *metafor* (v2.0-0; Viechtbauer, 2010). We used the standardized mean difference (SMD) and the rma() function as the meta-analytic random-effect model with the Hedges estimator (Viechtbauer, 2010). We also performed some additional analyses to test for publication bias. Publication bias occurs when the mean effect size in the overall dataset generates different conclusions from those obtained when the mean effect size comes from a representative sample with reliable results (Koricheva et al., 2013). We used both funnel plots (*i.e.*, scatterplots of effect sizes against their variance) and Spearman rank correlations between the mean effect size and sample sizes to test for publication bias (Koricheva et al., 2013). In the absence of bias, the funnel plots should show symmetry around the mean effect size, and effect sizes should not correlate with sample sizes (Koricheva et al., 2013).

## Results

The literature search resulted in 1,475 publications, of which 61 papers published between January 1992 and August 2017 met our criteria. The full data set is deposited in the Dryad Digital Repository. The publications were reported in 41 different journals (see Supplementary Materials). Considering only the studies that experimentally manipulated water availability we found three types of experiments: (1) studies that reduced the percentage of moisture in the soil compared to the control (44%), (2) studies that reduced the amount of water given to the plant (28%), and (3) studies that deprived the plants of water for the length of the study (28%). Eleven studies manipulated drought and aboveground herbivory simultaneously, and only one study also investigated the effects of root herbivory. Herbivory studies included nine species of herbivores who engaged in chewing (seven studies), sap-sucking (five studies), and artificial mechanical damage (two studies). Three studies measured the indirect effect of drought, by means of volatiles, on parasitoids. Forty-one studies measured leaf traits, 18 measured traits of both leaves and roots, and two measured only root traits. In total, the studies we reviewed investigated the responses of 92 plant species or genotypes in 48 genera. Crop species were used in 42 studies, native species in 16 studies, an invasive species in two studies, and a medicinal plant in one study. Sixty-seven percent of the studies were conducted in greenhouses or growth chambers, and 33% were field experiments.

Water treatment explained a significant proportion of trait variation for almost all plant traits measured (**Table 1**). For most traits, funnel plots of effect sizes versus sample sizes indicated that, overall, few studies reported these traits and that those studies were biased towards smaller sample sizes (Supplementary Figure A1). Nevertheless, Spearman’s rank correlations did not show significant relationships between the mean effect size and the sample size for 17 of the 19 traits we quantified (Supplementary Table A1), which indicates that bias in the meta-analysis is mostly non-significant, and our results are reliable estimates.

**Table 1:** The model heterogeneity (Q*_M_*) for changes in plant traits under drought treatment alone, and under drought and herbivory treatments combined.

Treatment per plant part	Plant trait measured	Q*_M_*	df	*P*
**Drought**				
Leaf	Primary metabolites	2400	240	*<0.001*
	Secondary metabolites	966	222	*<0.001*
	N-Based compounds	55.2	20	*<0.001*
	C-Based compounds	911	201	*<0.001*
	Physical traits	10.6	7	0.2
	Hormone	107	14	*<0.001*
	Biomass	329	33	*<0.001*
	Nutrients	263	35	*<0.001*
Root	Primary metabolites	158	17	*<0.001*
	Secondary metabolites	143	13	*<0.001*
	Physical traits	22.1	9	*<0.01*
	Hormone	7.18	3	0.07
	Biomass	154	20	*<0.001*
	Nutrients	86.6	9	*<0.001*
**Drought x Herbivory**		
Leaf	Primary metabolites	1.84	2	0.4
	Secondary metabolites	242	93	*<0.001*
	Hormone	30.6	14	*<0.01*

*A significant Q_M_ (P < 0.05) indicates that the treatment explains a significant proportion of the variance among observations.*

### Growth and Physical Traits

**FIGURE 1:**
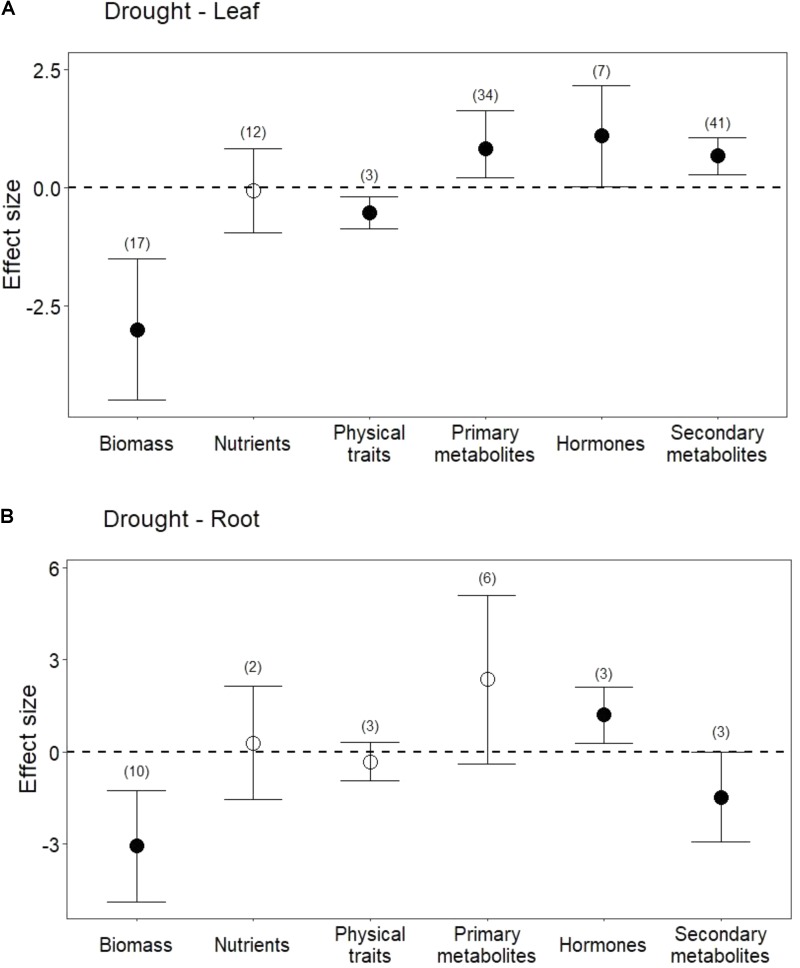
Influence of drought treatments on leaf and root traits. **(A)** Effect of drought on leaves. **(B)** Effect of drought on roots. Numbers in parentheses represent the number of studies considered. Mean effect sizes are shown with 95% confidence intervals (CIs). Effects are considered significant if their associated CIs do not overlap zero (dashed line) and are illustrated with solid circles.

Drought treatments reduced shoot and root biomass (**Figure 1**; *P* < 0.001 for both). Drought treatments also negatively affected physical traits (*i.e.*, lignin) in leaves (**Figure 1A**; *P* < 0.01). In contrast, drought did not change pooled physical traits in roots (*i.e*., lignin, and root length; **Figure 1B**; *P* = 0.3), but it did significantly reduce root length (Hedges’ *d* = -0.548, CI = -0.939 to -0.157, *P* < 0.01).

### Nutrients and Primary Metabolites

**FIGURE 2:**
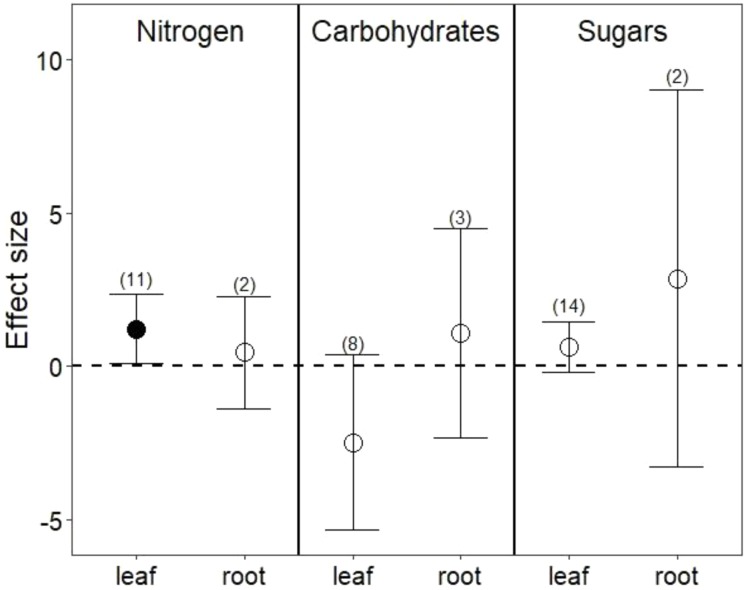
Drought effects on nitrogen content, complex carbohydrates, and sugars for leaves and roots. Numbers in parentheses represent the number of studies considered. Mean effect sizes are shown with 95% confidence intervals (CIs). Effects are considered significant if their associated CIs do not overlap zero (dashed line) and are illustrated with solid circles.

Drought treatments did not change overall nutrient content in leaves (**Figure 1A**; *P* = 0.8) or in roots (**Figure 1B**; *P* = 0.7). In leaves, this lack of effect emerged from the opposing effect of drought on nitrogen (N) compared with its effect on phosphorus (P) and potassium (K). Reduced water increased leaf N (**Figure 2**; *P* = 0.03) but reduced both P (Hedges’ *d* = -0.874, CI = -1.45 to -0.298, *P* < 0.01) and K (Hedges’ *d* = -2.798, CI = -4.111 to -1.485, *P* < 0.001). In contrast, drought did not affect root N (**Figure 2**; *P* = 0.6), and no data were available on changes in root P or K.

**FIGURE 3:**
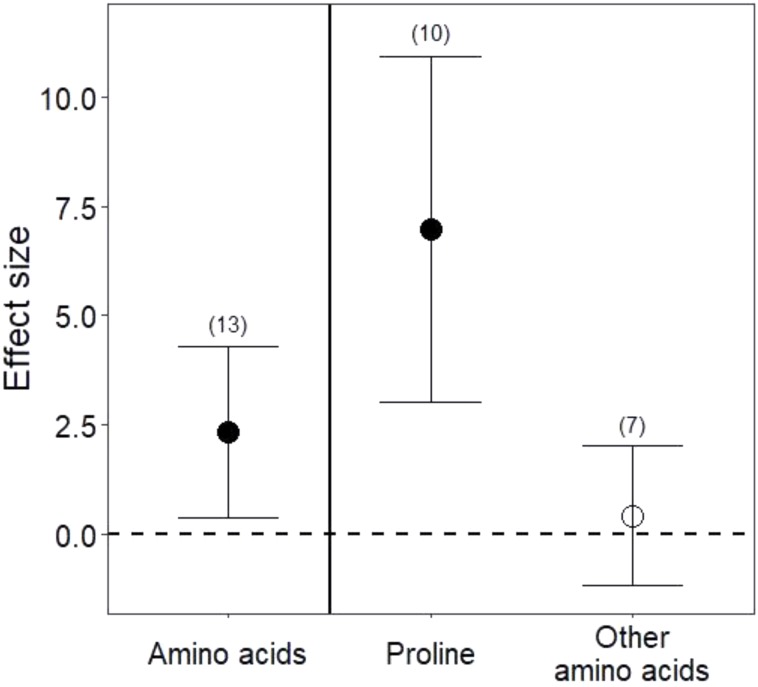
Drought effects on leaf amino acid content. “Amino acids” include all pooled amino acids; “Other amino acids” includes all amino acids except for proline. Numbers in parentheses represent the number of studies considered. Mean effect sizes are shown with 95% confidence intervals (CIs). Effects are considered significant if their associated CIs do not overlap zero (dashed line) and are illustrated with solid circles.

Drought treatments increased the concentrations of pooled primary metabolites in both leaves and roots, but this effect was significant only in leaves (**Figure 1**; *P* < 0.05 for leaves and *P* = 0.09 for roots). When primary metabolites were subdivided, drought treatments tended to increase sugars but reduce complex carbohydrates in leaves, but these effects were not significant (**Figure 2**). Very few studies examined sugars or complex carbohydrates in roots (**Figure 2**), and no studies examined amino acids or enzymes. In leaves, drought treatments had no effect on enzymes (*P* = 0.2) but did increase pooled amino acids (**Figure 3**; *P* < 0.03). Nevertheless, this increase in leaf amino acids depended almost entirely on proline (**Figure 3**).

## Hormones and Secondary Metabolites

Drought increased the concentrations of hormones (ABA and JA) in both leaves and roots (**Figure 1**; *P* < 0.05 and *P* < 0.01, respectively). On the other hand, salicylic acid (SA) was unchanged or marginally lower in leaves subjected to drought (Hedges’ *d* = -0.1015, CI = -1.007 to 0.803, *P* = 0.8).

**FIGURE 4:**
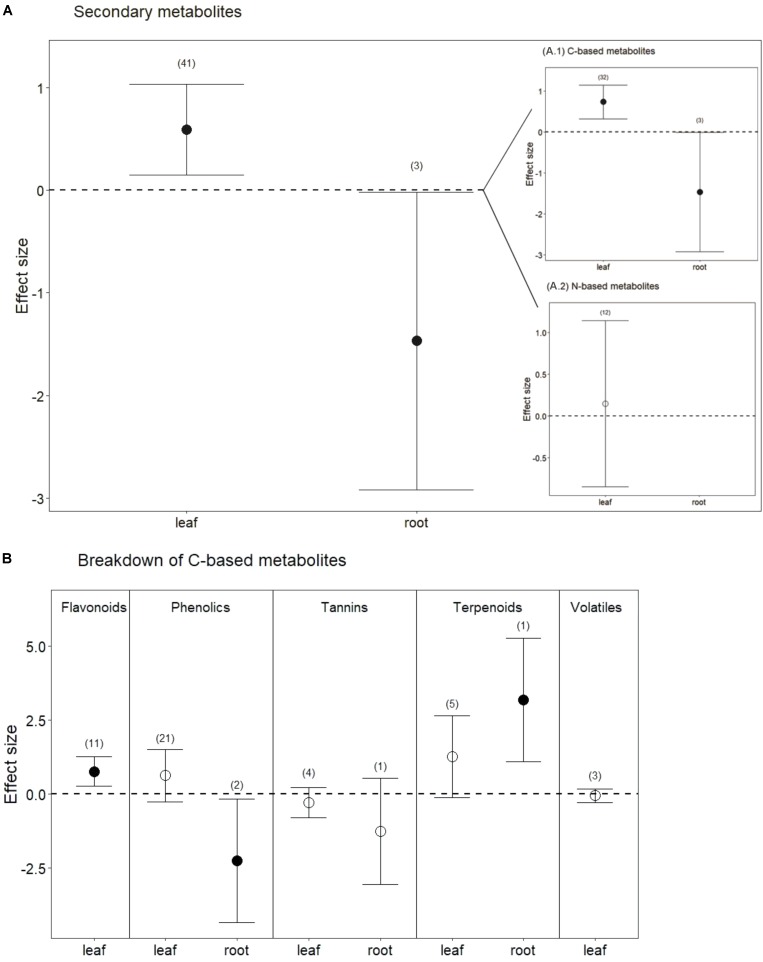
Secondary metabolite response to drought for both leaves and roots. **(A)** Secondary metabolites, including all measured metabolites (C-based and N-based) for each tissue (from **Figure 1**). **(A.1)** Carbon-based metabolites, including flavonoids, phenolics, tannins, terpenoids, and volatiles. **(A.2)** Nitrogen-based metabolites, including glucosinolates, alkaloids and glycoalkaloids. **(B)** Breakdown of C-based secondary metabolites for each tissue. Numbers in parentheses represent the number of studies considered. Mean effect sizes are shown with 95% confidence intervals (CIs). Effects are considered significant if their associated CIs do not overlap zero (dashed line) and are illustrated with solid circles.

Drought treatments also significantly increased secondary metabolites in leaves when all compounds were pooled (**Figures 1A**, **4**; *P* < 0.01). Interestingly, and in contrast to leaves, drought treatments significantly reduced secondary metabolites in roots (**Figures 1B**, **4**; *P* < 0.05). This difference between leaves and roots depended on carbon (C)-based secondary compounds, which were higher in leaves under drought but lower in roots (**Figure 4A.1**; *P* < 0.01 and *P* < 0.05, respectively). Drought did not affect nitrogen (N)-based secondary compounds in leaves (**Figure 4A.2**; *P* = 0.8), and these compounds were not measured in roots. Among C-based secondary metabolites, concentrations of flavonoids, phenolics, and terpenoids all increased in leaves under drought, but only the increase in flavonoids was significant (**Figure 4B**; *P* < 0.01). In contrast, phenolics in roots were significantly reduced by drought (**Figure 4B**; *P* < 0.04). Interestingly, although phenolics in leaves were not significantly higher under reduced water when all data were pooled, drought treatments significantly increased phenolics in non-trees (Hedges’ *d* = 1.953, CI = 0.191 to 3.715, *P* = 0.03), and marginally decreased phenolics in trees (Hedges’ *d* = -0.242, CI = -0.564 to 0.08, *P* = 0.1). Volatiles, mainly comprising monoterpenes and sesquiterpenes did not change in leaves under drought treatments (**Figure 4B**; *P* = 0.6), resulting from the opposing effects of two studies that found increases in volatiles and one that found a decrease. Volatiles under drought stress were not measured in roots.

### Herbivory and Tritrophic Interactions

**FIGURE 5:**
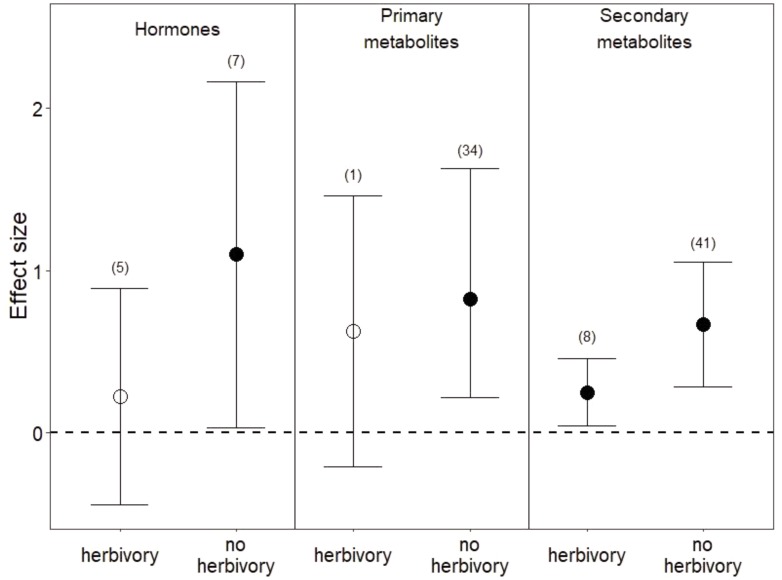
Effects of drought with and without herbivory on leaf metabolic responses. “No herbivory” effects are the same as those shown in **Figure 1** and are reproduced here for comparison. Numbers in parentheses represent the number of studies considered. Mean effect sizes are shown with 95% confidence intervals (CIs). Effects are considered significant if their associated CIs do not overlap zero (dashed line) and are illustrated with solid circles.

Among the 1,475 studies that were returned by our search criteria, only 11 studies manipulated water and herbivory simultaneously and contained sufficient observations for us to calculate an effect size. Overall, the co-occurrence of herbivory with reduced water appeared to dampen plant metabolic responses relative to those under reduced water alone (**Figure 5**). Hormones were slightly higher in leaves (**Figure 5**) and lower in roots in co-occurring drought and herbivory treatments relative to controls (*i.e.*, plants sustaining neither drought nor herbivory), but these effects were not significant (*P* = 0.5 in leaves and *P* = 0.2 in roots). Among studies that manipulated both drought and herbivory and also assessed root metabolic traits (Tariq et al., 2013a; Ederli et al., 2017), hormones were the only metabolite measured. In leaves, sugars were the only primary metabolites measured under co-occurring drought and herbivory treatments, and the slight increase in sugars was not significant (**Figure 5**; *P* = 0.1). Finally, co-occurring drought and herbivory treatments increased secondary metabolites in leaves when all compounds were pooled (**Figure 5**; *P* < 0.03), due to increases in C-based secondary metabolites (*P* < 0.04) and flavonoids in particular (*P* < 0.02). Still, the effect size of this increase in secondary metabolites under co-occurring drought and herbivory treatments was smaller than the increase observed under drought treatment alone (**Figure 5**).

The three studies that measured the effect of drought on volatiles also looked at parasitoid responses. These three studies illustrated a wide variety of possible outcomes: drought increased volatiles, which increased parasitoid attraction (Salerno et al., 2017); drought decreased volatiles, which decreased parasitoid attraction (Tariq et al., 2013b), and drought increased volatiles, but this had no effect on parasitoid attraction (Weldegergis et al., 2015).

## Discussion

Our results highlight the importance of considering whole-plant metabolic responses under water stress. Despite the key role of roots in sustaining and mitigating abiotic and biotic stress, early theories of trade-offs between plant growth and defense focused on aboveground tissues (Rhoades, 1979; Coley et al., 1985; Herms and Mattson, 1992). Although attention to roots has increased recently (*e.g.*, in our meta-analysis, a third of papers with root measurements were conducted since 2010), some metabolic comparisons between leaves and roots under water stress will be possible only with further studies. Additional studies will also be necessary to differentiate among levels of water stress, which could be associated with different plant metabolic responses. In the comparisons that were possible, leaves and roots often produced different metabolic responses, consistent with our first hypothesis. The leaf economics spectrum now provides simple predictions of how leaf traits should vary with resources, and such traits also correlate with vulnerability to herbivores (Wright et al., 2004; Agrawal and Fishbein, 2006). In contrast, an analogous root economics spectrum remains elusive (Valverde-Barrantes et al., 2015; Weemstra et al., 2016; also see Roumet et al., 2016), perhaps because roots respond to a wider variety of environmental constraints (Weemstra et al., 2016). Consistent with the existence of differential constraints operating above- and belowground, our results suggest that leaves and roots respond to water stress via partially decoupled growth-defense trade-offs.

Contrary to our second hypothesis, drought did not affect overall nutrient concentrations in either leaves or roots. However, this result obscured some interesting differences among nutrients, as well as between leaves and roots. Whereas P and K concentrations decreased in leaves under drought, N concentrations increased. This latter finding is in line with early work tying drought to increased N and higher herbivore pressure on aboveground tissues (*e.g.*, White, 1969), and it may result from studies where either the duration of drying or the frequency of rewetting was relatively high (He and Dijkstra, 2014). Surprisingly, a recent meta-analysis of N and P responses to drought found that soil extractable N, unlike P, can actually increase under drought treatment (He and Dijkstra, 2014; but see Delgado-Baquerizo et al., 2013). Roots, in contrast to leaves, showed no discernible changes in N concentration under drought, but only two studies were available for analysis.

Consistent with our third hypothesis, we found that some primary metabolites increased in both leaves and roots under drought. Plant growth is particularly sensitive to drought stress (Shao et al., 2008; Ings et al., 2013), and slowed growth may immediately redirect primary metabolism to the production of stress metabolites, such as sugars and free amino acids (Chaves et al., 2003). Reduced photosynthesis under drought also profoundly alters primary metabolism (Bradford and Hsiao, 1982; Lawlor, 2002). We found higher amino acids in leaves under drought stress, although this result was driven largely by increases in proline, an amino acid well known to adjust osmotic pressure, scavenge free radicals, and increase expression of stress-related genes under drought (Mali and Mehta, 1977; Chaves et al., 2003; Hayat et al., 2012). Among the studies we analyzed (Appendix 1), only one measured proline in the roots, but it too found that concentrations of proline nearly doubled under drought (El Sayed, 1992). These increases in free amino acids could be advantageous to nitrogen-limited herbivores (White, 1969), but proline metabolism has also been implicated in the production of plant phenolic secondary metabolites (Lattanzio et al., 2009), such that gains in accessible nitrogen to herbivores under drought may commonly be offset by higher plant toxicity (Gershenzon, 1984 and see below). Although sugars also marginally increased under drought in both leaves and roots (**Figure 2**), roots and leaves may metabolize complex carbohydrates at different rates under drought stress (**Figure 2**; Chaves, 1991). Because root:shoot biomass ratios increase under water stress (Poorter et al., 2012; Eziz et al., 2017), implying differential primary metabolism in below- and aboveground organs, future work quantifying primary metabolites in whole plants will be useful for determining whether and how primary metabolic responses reflect the source of stress and its relationship to organ function (*e.g.*, Merewitz et al., 2011; Tardieu, 2012; Gargallo-Garriga et al., 2014).

One of the main criteria of our meta-analysis was to find studies that measured secondary metabolites under water stress. Consistent with our fourth hypothesis, our results showed that concentrations of C-based secondary metabolites increased in leaves but decreased in roots under drought treatments. The effects of drought were also associated with plant type (tree or non-tree) and compound class. Phenolics were the most commonly measured secondary metabolites in drought studies. In all plants but trees, leaf phenolics increased whereas root phenolics decreased in response to drought. Although leaf phenolics in trees marginally decreased in response to drought, we did not find any studies that had measured root phenolics in trees for comparison. Increases in phenolics under water stress may be explained partly by their function as antioxidants (Nakabayashi et al., 2014), a function that may in fact be less necessary in roots than in leaves, because roots lack the spikes in reactive oxygen species under stress that are associated with chloroplasts (*e.g.*, Lodeyro et al., 2016). Many important secondary metabolites (e.g., alkaloids and flavonoids) known to be produced in roots (van Dam, 2009) were not measured in studies considering the effects of water stress. It will be important to consider a broader spectrum of root secondary metabolites and their responses to water stress in future studies. For example, flavonoids might be expected to increase in both roots and leaves under drought because these compounds can be enhanced by ABA signaling and modulate plant growth (Brown et al., 2001; Besseau et al., 2007).

Although water and herbivory stress frequently co-occur belowground (Bardgett and Wardle, 2003; Ryalls et al., 2016) and roots play a vital role in plant metabolism and fitness (Zangerl and Bazzaz, 1992; Lambers et al., 2002), growth-defense trade-offs and patterns of optimal defense are rarely determined for roots (van Dam, 2009). If the plant is integrating its responses above- and belowground under water stress, and if water stress increases the relative value of roots because they determine whole-plant water availability (Eziz et al., 2017), the plant might be expected to reduce its chemical defense of leaves and increase its chemical defense of roots. Our results, however, revealed the opposite pattern (**Figure 1**; see also Gargallo-Garriga et al., 2014). Notably, our results are consistent with the model of Zangerl and Bazzaz (1992), who argued that a higher root:shoot ratio under resource stress makes the roots comparatively *less* valuable per unit of structural investment. We propose that, at the level of the whole plant, there could be organ-specific variation in the optimal growth-defense ratio that is determined by the cost of the stress for the tissue and the tissue’s function for the plant (see also Freschet et al., 2013). Plants may invest in tolerance and regrowth of roots under drought stress, perhaps partly to explore a larger area of soil for water (*e.g.*, Hodge, 2004). In contrast, producing new leaves under drought conditions may be particularly expensive, leading to higher investment in aboveground chemical defenses. More information is needed to evaluate whole-plant growth-defense trade-offs under water stress, particularly because some important metabolic comparisons require additional root measurements. Addressing the trade-off only aboveground is problematic because, in addition to the stress responses of the roots themselves, root responses will influence leaf responses, as well as play a significant role in determining plant fitness and yield (*e.g.*, Bardgett and Wardle, 2003; War et al., 2012; Tariq et al., 2013a; Gargallo-Garriga et al., 2014).

Plants are known for their remarkable ability to respond to multiple stress conditions, sometimes using the same signaling pathway (Rizhsky et al., 2004; Fujita et al., 2006; Ramakrishna and Ravishankar, 2011). Our results are consistent with evidence that drought and herbivory may be regulated cooperatively by ABA/JA signaling. However, contrary to our fifth hypothesis, our results also suggest that the responses of secondary metabolites under co-occurring herbivory and drought were smaller than those under drought alone. Although this could be due to the smaller sample size of studies addressing the two stresses simultaneously, it is also possible that the combined energetic costs of drought and herbivory reduce the availability of energy and chemical precursors for an effective stress response (*e.g.*, Rennenberg et al., 2006; Zhu, 2016). Determining whether such costs are additive or synergistic should be a key goal for future research (*e.g.*, Bansal et al., 2013; Ben Rejeb et al., 2014).

Herbivores are strongly influenced not only from the bottom up by plant metabolites, but also from the top down by interactions with predators. Studies of tritrophic interactions in the context of drought stress typically examine only the population dynamics of herbivores and their natural enemies (Tariq et al., 2013b; Weldegergis et al., 2015; Salerno et al., 2017; see, *e.g.*, Hoover and Newman, 2004; Ahmed et al., 2017). Studies examining plant chemistry will be necessary to add predictive power to these, frequently context-dependent, observations. For example, plant metabolites can influence the effectiveness of the herbivore immune system, physical barriers against entomopathogens, and sequestration of secondary metabolites (War et al., 2012; Biere and Bennett, 2013; Duisembecov et al., 2017). Plant metabolites can also have differential effects on specialist and generalist herbivores (Ali and Agrawal, 2012), which could further modify community-wide food webs. We found only three studies of the indirect effects of drought on parasitoid predators via plant chemistry, and the results were inconsistent. Moreover, combining drought stress with herbivory stress may have additional important, non-additive effects on plant chemistry, and thus on the multitrophic interactions mediated by the plant (Shikano, 2017; Tariq et al., 2013b). Because plants are powerful mediators of interactions between otherwise loosely connected food webs, understanding phytochemical responses to co-occurring stresses will be crucial to predicting how terrestrial ecosystems will respond to global change.

## Conclusion

Drought and herbivory are common and important stresses in terrestrial ecosystems that can cause whole-plant changes in growth, physiology, and biochemistry. Whereas specific primary metabolites and changes in biomass are often measured in studies of drought and secondary metabolites are often measured in studies of herbivory, assessment of changes in a variety of primary and secondary metabolites, as well as physical traits, would lend insight into the complex metabolic and structural demands required for plants to acclimate and maintain function when faced with multiple stresses. Recent studies of plant- herbivore interactions clearly demonstrate that metabolic profiles of shoots can be altered by root herbivory and vice versa (Erb et al., 2008; Kaplan et al., 2008; Wondafrash et al., 2013), but it remains surprisingly rare to examine whole-plant responses to herbivory under co-occurring abiotic stress. Allocation to metabolic processes to tolerate and/or protect tissues from damage under stress can impact plant fitness and competitive ability, as well as plant mediation of multi-species trophic interactions. Prediction of the effects of multiple stresses on plant metabolic allocation and its ecological ramifications will ultimately require a theoretical framework for the whole plant.

## Author Contributions

FM and EP contributed to the design and implementation of the study, to the analysis of the results, and to the writing of the manuscript.

## Conflict of Interest Statement

The authors declare that the research was conducted in the absence of any commercial or financial relationships that could be construed as a potential conflict of interest.

## Supplementary Material

All of the data and R code for the figures and supplementary material are deposited in the Dryad Digital Repository.
