# Supplementary figures and images for: Whole-Plant Metabolic Allocation Under Water Stress
Source: Front Plant Sci. 2018 Jun 25;9:852. doi: 10.3389/fpls.2018.00852 (PMC6026660; doi:10.3389/fpls.2018.00852)

Figure A1

Drought

A. Leaf

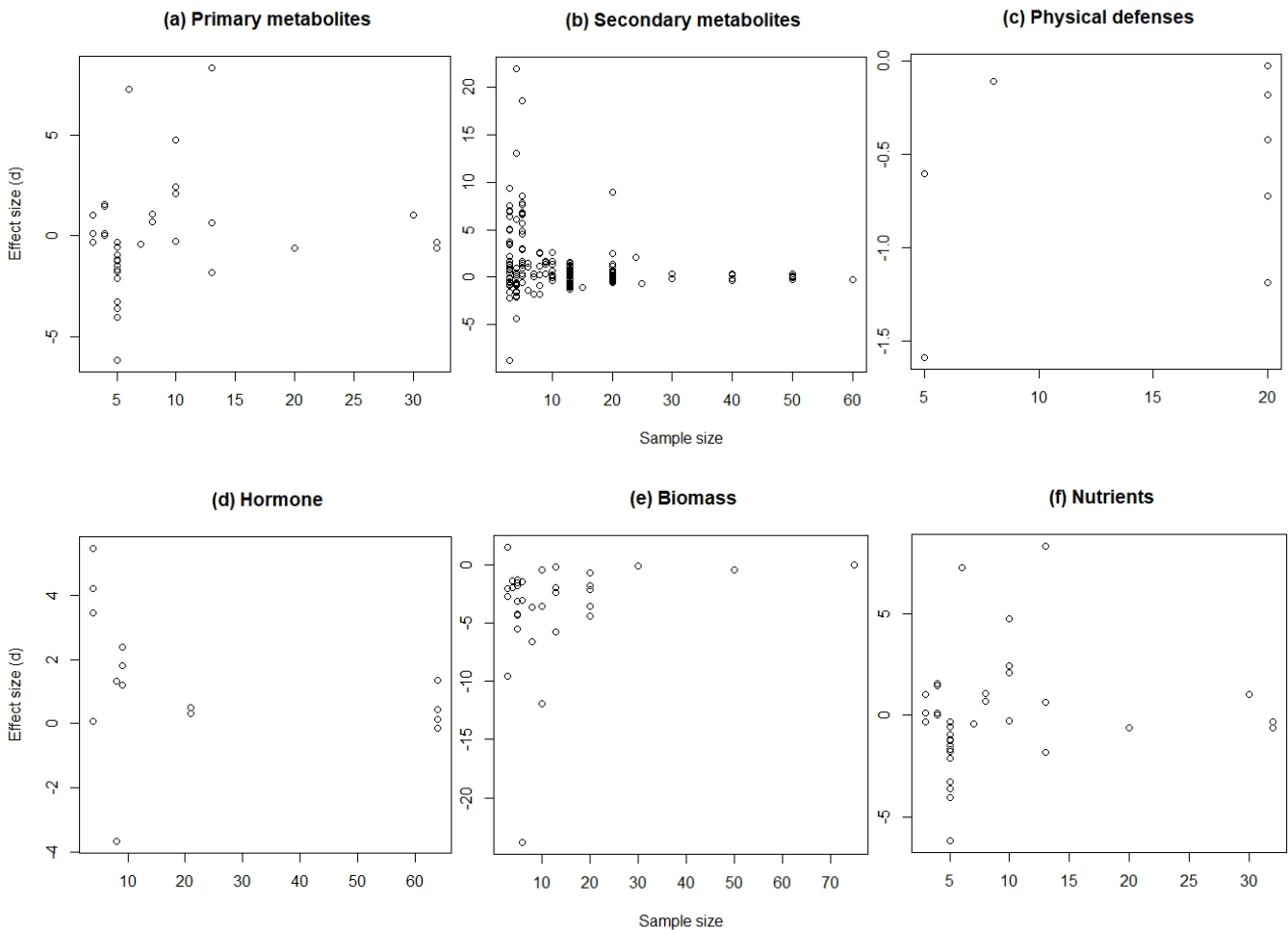

# Drought

## B. Root

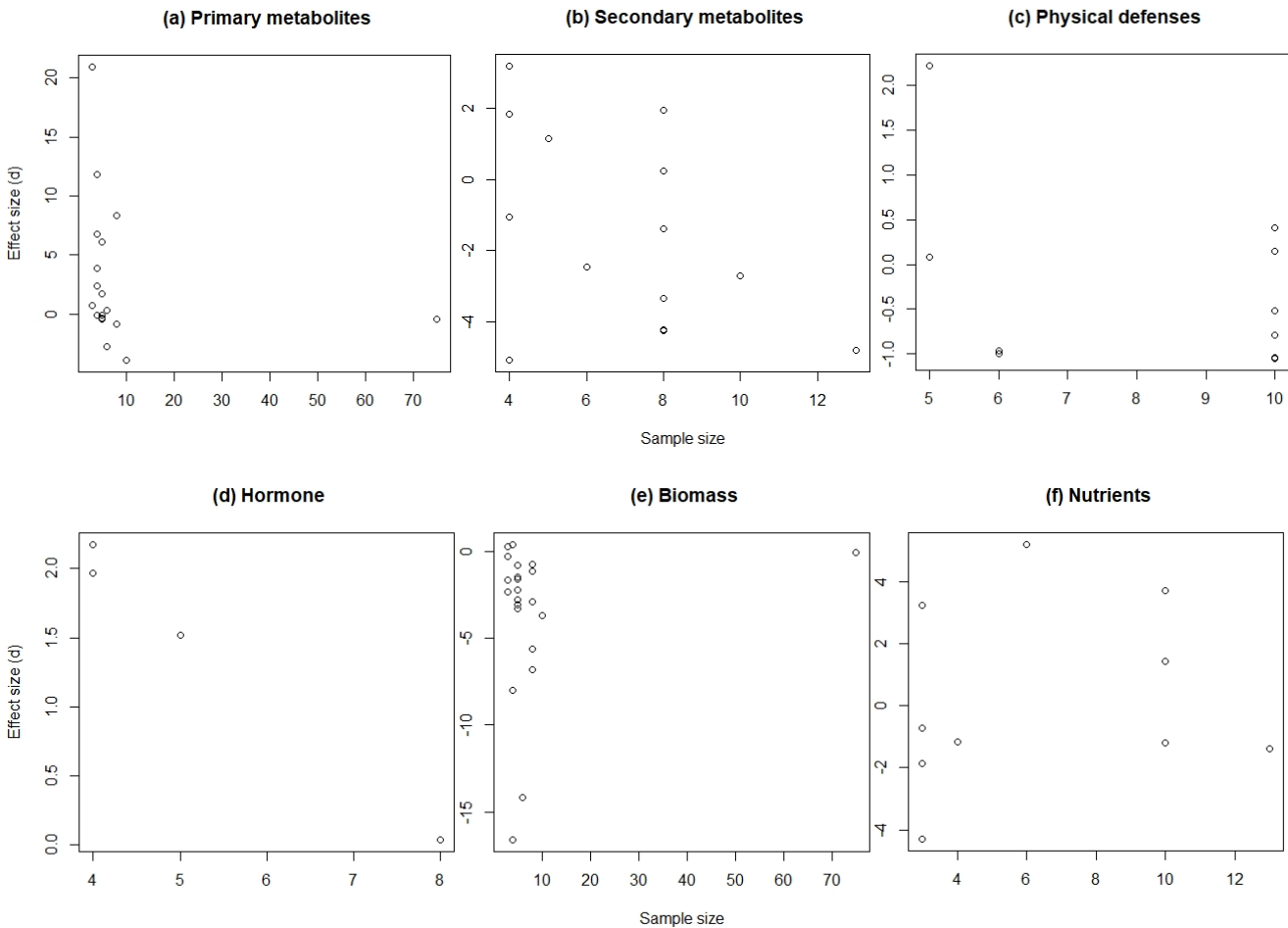

### C. Drought x Herbivory

#### Leaf

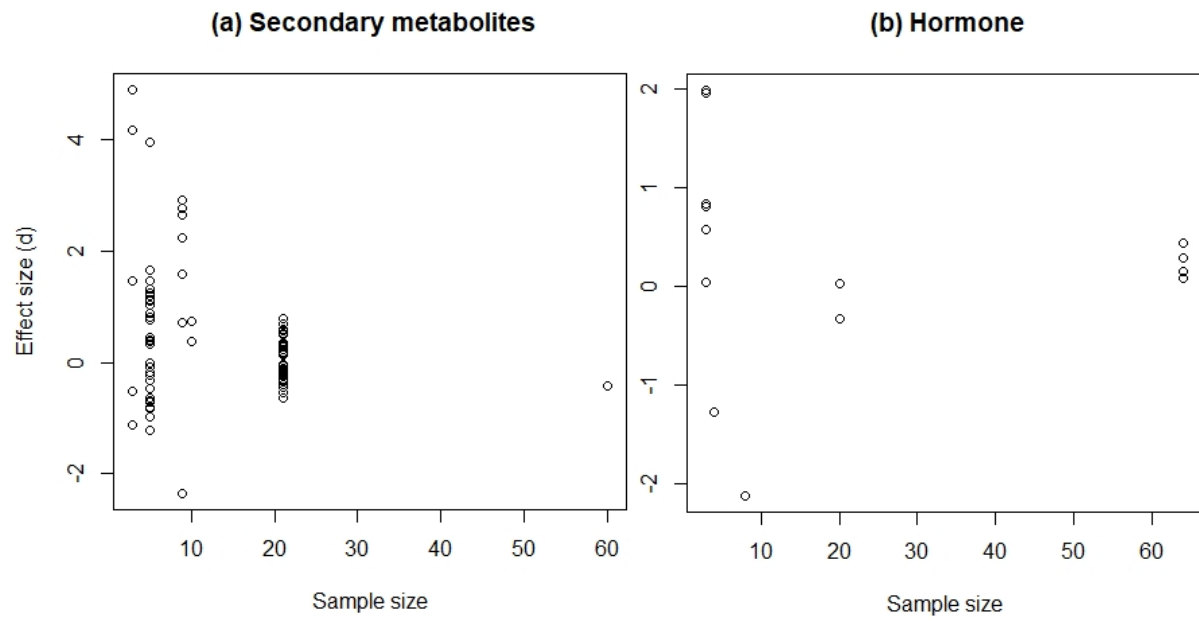

Supplement: Supplementary file 1 [file Image_1.pdf]
