# Supplementary material for: Whole-Plant Metabolic Allocation Under Water Stress
Source: Front Plant Sci. 2018 Jun 25;9:852. doi: 10.3389/fpls.2018.00852 (PMC6026660; doi:10.3389/fpls.2018.00852)
Supplement: Supplementary file 2 [file Table_1.docx]

**Table A1.** Spearman rank correlation test for symmetry. Non-significant (P>0.05) correlations support the lack of publication bias.

| Treatment per plant part | Plant trait measured | r | P |
| --- | --- | --- | --- |
| *Drought* |  |  |  |
| Leaf | Primary metabolites | 2470000 | 0.4 |
|  | Secondary metabolites |  |  |
|  | C-based |  |  |
|  | Flavonoids | 17800 | 0.8 |
|  | Phenolics | 43700 | 0.7 |
|  | Tannins | 308 | 0.8 |
|  | Terpenoids | 107 | 0.5 |
|  | Volatiles | 41800 | **0.001** |
|  | N-based | 1880 | 0.3 |
|  | Physical defenses | 59.8 | 0.5 |
|  | Hormone | 809 | 0.1 |
|  | Biomass | 5450 | 0.3 |
|  | Nutrients | 6500 | 0.3 |
|  |  |  |  |
| Root | Primary metabolites | 1560 | **0.007** |
|  | Secondary metabolites | 636 | 0.2 |
|  | Physical defenses | 210. | 0.4 |
|  | Hormone | 19.5 | 0.06 |
|  | Biomass | 2080 | 0.4 |
|  | Nutrients | 134 | 0.6 |
|  |  |  |  |
| *Drought x herbivory* | |  |  |
| Leaf | |  |  |
|  | Primary metabolites | NA | NA |
|  | Secondary metabolites | 167000 | 0.05 |
|  | Hormone | 770. | 0.2 |
