# Supplementary material for: Whole-Plant Metabolic Allocation Under Water Stress
Source: Front Plant Sci. 2018 Jun 25;9:852. doi: 10.3389/fpls.2018.00852 (PMC6026660; doi:10.3389/fpls.2018.00852)
Supplement: Supplementary file 3 [file Data_Sheet_1.docx]

Appendix A1

References used in the meta-analysis

Ahmed, S., Orians, C. M., Griffin, T. S., Buckley, S., Unachukwu, U., Stratton, A. E., et al. (2014). Effects of water availability and pest pressures on tea (*Camellia sinensis*) growth and functional quality. *Aob Plants* 6, plt054. doi:10.1093/aobpla/plt054.

Ali, Q., Haider, M. Z., Iftikhar, W., Jamil, S., Javed, M. T., Noman, A., et al. (2016). Drought tolerance potential of *Vigna mungo* L. lines as deciphered by modulated growth, antioxidant defense, and nutrient acquisition patterns. *Braz. J. Bot.* 39, 801–812. doi:10.1007/s40415-016-0282-y.

Aninbon, C., Jogloy, S., Vorasoot, N., Patanothai, A., Nuchadomrong, S., and Senawong, T. (2016). Effect of end of season water deficit on phenolic compounds in peanut genotypes with different levels of resistance to drought. *Food Chem.* 196, 123–129. doi:10.1016/j.foodchem.2015.09.022.

Anjum, S. A., Farooq, M., Xie, X., Liu, X., and Ijaz, M. F. (2012). Antioxidant defense system and proline accumulation enables hot pepper to perform better under drought. *Sci. Hortic.* 140, 66–73. doi:10.1016/j.scienta.2012.03.028.

Caser, M., D’Angiolillo, F., Chitarra, W., Lovisolo, C., Ruffoni, B., Pistelli, L., et al. (2016). Water deficit regimes trigger changes in valuable physiological and phytochemical parameters in *Helichrysum petiolare* Hilliard & BL Burtt. *Ind. Crops Prod.* 83, 680–692. doi:10.1016/j.indcrop.2015.12.053.

Casteel, C. L., Niziolek, O. K., Leakey, A. D. B., Berenbaum, M. R., and DeLucia, E. H. (2012). Effects of elevated CO2 and soil water content on phytohormone transcript induction in *Glycine max* after *Popillia japonica* feeding. *Arthropod-Plant Interact.* 6, 439–447. doi:10.1007/s11829-012-9195-2.

Cella Pizarro, L., and Bisigato, A. J. (2010). Allocation of biomass and photoassimilates in juvenile plants of six Patagonian species in response to five water supply regimes. *Ann. Bot.* 106, 297–307. doi:10.1093/aob/mcq109.

Cipollini, M. L., Paulk, E., and Cipollini, D. F. (2002). Effect of nitrogen and water treatment on leaf chemistry in horsenettle (*Solanum carolinense*), and relationship to herbivory by flea beetles (*Epitrix* spp.) and tobacco hornworm (*Manduca sexta*). *J. Chem. Ecol.* 28, 2377–2398. doi:10.1023/A:1021494315786.

Copolovici, L., Kaennaste, A., Remmel, T., and Niinemets, U. (2014). Volatile organic compound emissions from *Alnus glutinosa* under interacting drought and herbivory stresses. *Environ. Exp. Bot.* 100, 55–63. doi:10.1016/j.envexpbot.2013.12.011.

Ederli, L., Brunetti, C., Centritto, M., Colazza, S., Frati, F., Loreto, F., et al. (2017). Infestation of Broad Bean (*Vicia faba*) by the Green Stink Bug (*Nezara viridula)* Decreases Shoot Abscisic Acid Contents under Well-Watered and Drought Conditions. *Front. Plant Sci.* 8, 959. doi:10.3389/fpls.2017.00959.

Eichholz, I., Foerster, N., Ulrichs, C., Schreiner, M., and Huyskens-Keil, S. (2014). Survey of bioactive metabolites in selected cultivars and varieties of *Lactuca sativa* L. under water stress. *J. Appl. Bot. Food Qual.* 87, 265–273. doi:10.5073/JABFQ.2014.087.037.

el Sayed, H. (1992). Proline metabolism during water-stress in sweet-pepper (*Capsicum annuum* L.) plant. *Phyton-Ann. Rei Bot.* 32, 255–261.

EnglishLoeb, G., Stout, M. J., and Duffey, S. S. (1997). Drought stress in tomatoes: Changes in plant chemistry and potential nonlinear consequences for insect herbivores. *Oikos* 79, 456–468. doi:10.2307/3546888.

Estiarte, M., Filella, I., Serra, J., and Peñuelas, J. (1994). Effects of nutrient and water stress on leaf phenolic content of peppers and susceptibility to generalist herbivore *Helicoverpa armigera* (Hubner). *Oecologia* 99, 387–391. doi:10.1007/BF00627753.

Fernandez de Simon, B., Sanz, M., Teresa Cervera, M., Pinto, E., Aranda, I., and Cadahia, E. (2017). Leaf metabolic response to water deficit in *Pinus pinaster* Ait. relies upon ontogeny and genotype. *Environ. Exp. Bot.* 140, 41–55. doi:10.1016/j.envexpbot.2017.05.017.

Gleadow, R. M., and Woodrow, I. E. (2002). Defense chemistry of cyanogenic *Eucalyptus cladocalyx* seedlings is affected by water supply. *Tree Physiol.* 22, 939–945. doi:10.1093/treephys/22.13.939.

Glynn, C., Ronnberg-Wastljung, A. C., Julkunen-Tiitto, R., and Weih, M. (2004). Willow genotype, but not drought treatment, affects foliar phenolic concentrations and leaf-beetle resistance. *Entomol. Exp. Appl.* 113, 1–14. doi:10.1111/j.0013-8703.2004.00199.x.

Guo, H., Sun, Y., Peng, X., Wang, Q., Harris, M., and Ge, F. (2016). Up-regulation of abscisic acid signaling pathway facilitates aphid xylem absorption and osmoregulation under drought stress. *J. Exp. Bot.* 67, 681–693. doi:10.1093/jxb/erv481.

Gutbrodt, B., Dorn, S., and Mody, K. (2012a). Drought stress affects constitutive but not induced herbivore resistance in apple plants. *Arthropod-Plant Interact.* 6, 171–179. doi:10.1007/s11829-011-9173-0.

Gutbrodt, B., Dorn, S., Unsicker, S. B., and Mody, K. (2012b). Species-specific responses of herbivores to within-plant and environmentally mediated between-plant variability in plant chemistry. *Chemoecology* 22, 101–111. doi:10.1007/s00049-012-0102-1.

Han, P., Wang, Z., Lavoir, A.-V., Michel, T., Seassau, A., Zheng, W., et al. (2016). Increased water salinity applied to tomato plants accelerates the development of the leaf miner *Tuta absoluta* through bottom-up effects. *Sci. Rep.* 6, 32403. doi:10.1038/srep32403.

Hassan, N., El-Bastawisy, Z., Ebeed, H., and Alla, M. N. (2015). Role of defense enzymes, proteins, solutes and Delta 1-pyrroline-5-carboxylate synthase in wheat tolerance to drought. *Rendiconti Lincei-Sci. Fis. E Nat.* 26, 281–291. doi:10.1007/s12210-015-0429-y.

Jamieson, M. A., Quintero, C., and Blumenthal, D. M. (2013). Interactive effects of simulated nitrogen deposition and altered precipitation patterns on plant allelochemical concentrations. *J. Chem. Ecol.* 39, 1204–1208. doi:10.1007/s10886-013-0340-x.

Karageorgou, P., Levizou, E., and Manetas, Y. (2002). The influence of drought, shade and availability of mineral nutrients on exudate phenolics of *Dittrichia viscosa*. *Flora* 197, 285–289. doi:10.1078/0367-2530-00042.

Keles, Y., and Oncel, I. (2002). Response of antioxidative defence system to temperature and water stress combinations in wheat seedlings. *Plant Sci.* 163, 783–790. doi:10.1016/S0168-9452(02)00213-3.

Keles, Y., and Unyayar, S. (2004). Responses of antioxidant defense system of *Helianthus annuus* to abscisic acid treatment under drought and waterlogging. *Acta Physiol. Plant.* 26, 149–156. doi:10.1007/s11738-004-0004-0.

Khan, M. A. M., Ulrichs, C., and Mewis, I. (2010). Influence of water stress on the glucosinolate profile of *Brassica oleracea* var. *italica* and the performance of *Brevicoryne brassicae* and *Myzus persicae*. *Entomol. Exp. Appl.* 137, 229–236. doi:10.1111/j.1570-7458.2010.01059.x.

Khan, M. A. M., Ulrichs, C., and Mewis, I. (2011). Effect of water stress and aphid herbivory on flavonoids in broccoli (*Brassica oleracea* var. *italica* Plenck). *J. Appl. Bot. Food Qual.-Angew. Bot.* 84, 178–182.

Kosar, F., Akram, N. A., and Ashraf, M. (2015). Exogenously-applied 5-aminolevulinic acid modulates some key physiological characteristics and antioxidative defense system in spring wheat (*Triticum aestivum* L.) seedlings under water stress. *South Afr. J. Bot.* 96, 71–77. doi:10.1016/j.sajb.2014.10.015.

Lama, A. D., Kim, J., Martiskainen, O., Klemola, T., Salminen, J.-P., Tyystjarvi, E., et al. (2016). Impacts of simulated drought stress and artificial damage on concentrations of flavonoids in *Jatropha curcas* (L.), a biofuel shrub. *J. Plant Res.* 129, 1141–1150. doi:10.1007/s10265-016-0850-z.

Lavinsky, A. O., Magalhaes, P. C., Avila, R. G., Diniz, M. M., and de Souza, T. C. (2015). Partitioning between primary and secondary metabolism of carbon allocated to roots in four maize genotypes under water deficit and its effects on productivity. *Crop J.* 3, 379–386. doi:10.1016/j.cj.2015.04.008.

Liu, B., Lei, C., Jin, J., Li, S., Zhang, Y., and Liu, W. (2015). Physiological responses of two moss species to the combined stress of water deficit and elevated nitrogen deposition. I. Secondary metabolism. *Int. J. Plant Sci.* 176, 446–457. doi:10.1086/681023.

Liu, C., Wang, Y., Pan, K., Li, W., Zhang, L., Shen, X., et al. (2014). Responses of the antioxidant defense system to drought stress in the leaves of *Fargesia denudata* seedlings, the staple food of the giant panda. *Russ. J. Plant Physiol.* 61, 374–383. doi:10.1134/S1021443714020083.

Malik, N. S. A., Perez, J. L., Kunta, M., Patt, J. M., and Mangan, R. L. (2014). Changes in free amino acids and polyamine levels in *Satsuma* leaves in response to *Asian citrus* psyllid infestation and water stress. *Insect Sci.* 21, 707–716. doi:10.1111/1744-7917.12075.

McKiernan, A. B., Hovenden, M. J., Brodribb, T. J., Potts, B. M., Davies, N. W., and O’Reilly-Wapstra, J. M. (2014). Effect of limited water availability on foliar plant secondary metabolites of two *Eucalyptus* species. *Environ. Exp. Bot.* 105, 55–64. doi:10.1016/j.envexpbot.2014.04.008.

McKiernan, A. B., Potts, B. M., Brodribb, T. J., Hovenden, M. J., Davies, N. W., McAdam, S. A. M., et al. (2016). Responses to mild water deficit and rewatering differ among secondary metabolites but are similar among provenances within *Eucalyptus* species. *Tree Physiol.* 36, 133–147. doi:10.1093/treephys/tpv106.

Nguyen, D., D’Agostino, N., Tytgat, T. O. G., Sun, P., Lortzing, T., Visser, E. J. W., et al. (2016). Drought and flooding have distinct effects on herbivore-induced responses and resistance in *Solanum dulcamara*. *Plant Cell Environ.* 39, 1485–1499. doi:10.1111/pce.12708.

Niu, Y., Wang, Y., Li, P., Zhang, F., Liu, H., and Zheng, G. (2013). Drought stress induces oxidative stress and the antioxidant defense system in ascorbate-deficient vtc1 mutants of *Arabidopsis thaliana*. *Acta Physiol. Plant.* 35, 1189–1200. doi:10.1007/s11738-012-1158-9.

Patade, V. Y., Bhargava, S., and Suprasanna, P. (2011). Salt and drought tolerance of sugarcane under iso-osmotic salt and water stress: growth, osmolytes accumulation, and antioxidant defense. *J. Plant Interact.* 6, 275–282. doi:10.1080/17429145.2011.557513.

Pineda, A., Pangesti, N., Soler, R., van Dam, N. M., van Loon, J. J. A., and Dicke, M. (2016). Negative impact of drought stress on a generalist leaf chewer and a phloem feeder is associated with, but not explained by an increase in herbivore-induced indole glucosinolates. *Environ. Exp. Bot.* 123, 88–97. doi:10.1016/j.envexpbot.2015.11.007.

Prill, N., Bullock, J. M., van Dam, N. M., and Leimu, R. (2014). Loss of heterosis and family-dependent inbreeding depression in plant performance and resistance against multiple herbivores under drought stress. *J. Ecol.* 102, 1497–1505. doi:10.1111/1365-2745.12327.

Rani, P. U., and Prasannalaxmi, K. (2014). Water stress induced physiological and biochemical changes in *Piper betle* L. and *Ricinus communis* L. plants and their effects on *Spodoptera litura*. *Allelopathy J.* 33, 25–41.

Roth, S., McDonald, E. P., and Lindroth, R. L. (1997). Atmospheric CO2 and soil water availability: consequences for tree-insect interactions. *Can. J. For. Res.* 27, 1281–1290.

Shafiq, S., Akram, N. A., Ashraf, M., and Arshad, A. (2014). Synergistic effects of drought and ascorbic acid on growth, mineral nutrients and oxidative defense system in canola (*Brassica napus* L.) plants. *Acta Physiol. Plant.* 36, 1539–1553. doi:10.1007/s11738-014-1530-z.

Song, F., Han, X., Zhu, X., and Herbert, S. J. (2012). Response to water stress of soil enzymes and root exudates from drought and non-drought tolerant corn hybrids at different growth stages. *Can. J. Soil Sci.* 92, 501–507. doi:10.4141/CJSS2010-057.

Soni, P., and Abdin, M. Z. (2017). Water deficit-induced oxidative stress affects artemisinin content and expression of proline metabolic genes in *Artemisia annua* L. *Febs Open Bio* 7, 367–381. doi:10.1002/2211-5463.12184.

Steinbrenner, A. D., Agerbirk, N., Orians, C. M., and Chew, F. S. (2012). Transient abiotic stresses lead to latent defense and reproductive responses over the *Brassica rapa* life cycle. *Chemoecology* 22, 239–250. doi:10.1007/s00049-012-0113-y.

Tanase, C., Boz, I., Stingu, A., Volf, I., and Popa, V. I. (2014). Physiological and biochemical responses induced by spruce bark aqueous extract and deuterium depleted water with synergistic action in sunflower (*Helianthus annuus* L.) plants. *Ind. Crops Prod.* 60, 160–167. doi:10.1016/j.indcrop.2014.05.039.

Tariq, M., Rossiter, J. T., Wright, D. J., and Staley, J. T. (2013a). Drought alters interactions between root and foliar herbivores. *Oecologia* 172, 1095–1104. doi:10.1007/s00442-012-2572-9.

Tariq, M., Wright, D. J., Bruce, T. J. A., and Staley, J. T. (2013b). Drought and root herbivory interact to alter the response of above-ground parasitoids to aphid infested plants and associated plant volatile signals. *Plos One* 8, e69013. doi:https://doi.org/10.1371/journal.pone.0069013.

Tattini, M., Loreto, F., Fini, A., Guidi, L., Brunetti, C., Velikova, V., et al. (2015). Isoprenoids and phenylpropanoids are part of the antioxidant defense orchestrated daily by drought-stressed *Platanus xacerifolia* plants during Mediterranean summers. *New Phytol.* 207, 613–626. doi:10.1111/nph.13380.

Vaughan, M. M., Huffaker, A., Schmelz, E. A., Dafoe, N. J., Christensen, S. A., McAuslane, H. J., et al. (2016). Interactive effects of elevated [CO2] and drought on the maize phytochemical defense response against *Mycotoxigenic fusarium* verticillioides. *Plos One* 11, e0159270. doi:10.1371/journal.pone.0159270.

Vilela, A. E., Agueero, P. R., Ravetta, D. A., and Gonzalez-Paleo, L. (2012). Long-term plasticity in growth, storage and defense allocation produces drought-tolerant juvenile shrubs of *Prosopis alpataco* RA Philippi (Fabaceae). *Flora* 207, 436–441. doi:10.1016/j.flora.2012.02.006.

Walter, J., Hein, R., Auge, H., Beierkuhnlein, C., Loeffler, S., Reifenrath, K., et al. (2012). How do extreme drought and plant community composition affect host plant metabolites and herbivore performance? *Arthropod-Plant Interact.* 6, 15–25. doi:10.1007/s11829-011-9157-0.

Weldegergis, B. T., Zhu, F., Poelman, E. H., and Dicke, M. (2015). Drought stress affects plant metabolites and herbivore preference but not host location by its parasitoids. *Oecologia* 177, 701–713. doi:10.1007/s00442-014-3129-x.

Wu, S., Hu, C., Tan, Q., Li, L., Shi, K., Zheng, Y., et al. (2015). Drought stress tolerance mediated by zinc-induced antioxidative defense and osmotic adjustment in cotton (*Gossypium Hirsutum*). *Acta Physiol. Plant.* 37, 167. doi:10.1007/s11738-015-1919-3.

Xiaolu, W., Jie, Y., Aoxue, L., Yu, C., and Yijun, F. (2016). Drought stress and re-watering increase secondary metabolites and enzyme activity in dendrobium moniliforme. *Ind. Crops Prod.* 94, 385–393. doi:10.1016/j.indcrop.2016.08.041.

Xu, Y., Xu, Q., and Huang, B. (2015). Ascorbic acid mitigation of water stress-inhibition of root growth in association with oxidative defense in tall fescue (*Festuca arundinacea* Schreb.). *Front. Plant Sci.* 6, 807. doi:10.3389/fpls.2015.00807.

Yang, J. C., Zhang, J. H., Wang, Z. Q., Zhu, Q. S., and Liu, L. J. (2002). Abscisic acid and cytokinins in the root exudates and leaves and their relationship to senescence and remobilization of carbon reserves in rice subjected to water stress during grain filling. *Planta* 215, 645–652. doi:10.1007/s00425-002-0789-2.

Zhang, M., Lv, D., Ge, P., Bian, Y., Chen, G., Zhu, G., et al. (2014). Phosphoproteome analysis reveals new drought response and defense mechanisms of seedling leaves in bread wheat (*Triticum aestivum* L.). *J. Proteomics* 109, 290–308. doi:10.1016/j.jprot.2014.07.010.
